# Molecular Survey and Phylogenetic Analysis of Atypical Porcine Pestivirus (APPV) Identified in Swine and Wild Boar from Northern Italy

**DOI:** 10.3390/v11121142

**Published:** 2019-12-10

**Authors:** Enrica Sozzi, Cristian Salogni, Davide Lelli, Ilaria Barbieri, Ana Moreno, Giovanni Loris Alborali, Antonio Lavazza

**Affiliations:** Istituto Zooprofilattico Sperimentale della Lombardia e dell’Emilia Romagna “Bruno Ubertini” (IZSLER), Via Antonio Bianchi 7/9, 25124 Brescia, Italy; cristian.salogni@izsler.it (C.S.); davide.lelli@izsler.it (D.L.); ilaria.barbieri@izsler.it (I.B.); anamaria.morenomartin@izsler.it (A.M.); giovanni.alborali@izsler.it (G.L.A.); antonio.lavazza@izsler.it (A.L.)

**Keywords:** pestivirus, pig, APPV, phylogenetic analysis, Italy

## Abstract

Atypical porcine pestivirus (APPV) is a newly recognized member of the *Flaviviridae* family. This novel porcine pestivirus was first described in 2015 in the USA, where it has been associated with congenital tremor type A-II in new-born piglets. APPV is widely distributed in domestic pigs in Europe and Asia. In this study, a virological survey was performed in Northern Italy to investigate the presence of APPV using molecular methods. Testing of 360 abortion samples from pig herds revealed two APPV strains from distinct provinces in the Lombardy region and testing of 430 wild boar blood samples revealed three strains, one from Lombardy and two from Emilia Romagna. The nucleotide sequencing of a fragment of the nonstructural protein 3-coding region revealed a high similarity to the previously detected European strains (Spanish, German, and Italian) of APPV.

## 1. Introduction

Pestiviruses are highly variable single-stranded RNA genome viruses, belonging to the *Flaviviridae* family. Actually, based on molecular and epidemiological evidence, the genus Pestivirus includes eleven species, indicated with progressive letters from A to K. Thus, the “classical” species are A (Bovine viral diarrhea virus 1), B (Bovine viral diarrhea virus 2), C (Classical swine fever virus), and D (Border disease virus), whereas the new species are E to K [1]. In addition, other pestiviruses were described, likely as three additional species, respectively, in bats (bat-derived pestivirus) [2,3], sheep and goats (Tunisian sheep pestiviruses) [4], and pigs (Linda pestivirus) [5]. In the swine species only, in addition to Pestivirus C, three pestiviruses have been identified to date: (1) Pestivirus F (Bungowannah virus) reported only in Australia, as a cause of reproductive disorders, fetal death, and sudden death in piglets, [6]; (2) The Linda virus described in association with congenital tremors (CTs) in piglets in Austria and thereafter only occasionally reported, leaving its geographical spread and clinical relevance in pigs undefined [5]; and (3) Pestivirus K, commonly known as atypical porcine pestivirus (APPV), which is the most relevant due to the frequency of identification, clinical findings, and economic importance. In fact, it has been identified several times in North America [7,8], South America [9,10], Europe [11,12,13], and Asia [14,15], and it should be considered as stably present for a long time in domestic and wild pig populations [8,10,15,16]. This is true also for Italy, since in a previous survey, at least four APPV isolates were found between 2015–2017 and a quite high seroprevalence was detected in pig sera [15].

Although APPV has been repeatedly identified in asymptomatic animals, there is clear evidence that it is associated with CT syndrome type A-II (CT A-II) in newborns [7]. Clinically healthy pigs and wild boars may have an epidemiological role as vehicles of APPV, but, considering the different frequency of detection in wild boars, which was quite high in Germany and Serbia [17] and very low in Spain [18], the epidemiology of APPV may vary considerably from country to country with increases in livestock and wild populations, animal breeding, and world trade [18]. While one study described the economic losses caused by a 10% drop in the number of weaned piglets per sow [19], the full economic consequences of APPV outbreaks remain to be determined.

In this study, the presence of the APPV genome in pig fetuses and wild boars from both the Lombardy and Emilia Romagna regions of Northern Italy was determined, and the genetic characterizations of the identified strains are described.

## 2. Materials and Methods

### 2.1. Pigs

From 2016 to 2018, 360 fetuses of pigs from pig farms in the Lombardy region were examined at the IZSLER Diagnostic Laboratory in Brescia. All of the samples examined originated from field cases of spontaneous abortions in pig farms and sent to the general diagnostic laboratory of IZSLER to determine the presence of any infectious agent. In none of these cases was the presence of clinical signs referable to CT syndrome specifically reported. During necropsy, samples of organs (brain, lung, spleen, liver, and kidney) were taken from each aborted fetus, then collected into a single farm-specific pool and homogenized (10% w/v) in minimum essential medium (MEM; Gibco, Life Technologies, Paisley, UK) supplemented with an antibiotic (1000 U/mL penicillin, 1 mg/mL streptomycin; Gibco, Life Technologies, Paisley, UK) and anti-mycotic (2.5 µg/mL amphotericin B; Gibco, Life Technologies, Paisley, UK). After centrifugation, the supernatant was analyzed to identify any agents that cause abortions in swine. For bacteriological agents such as Brucella spp., Listeria spp., and Mycoplasma spp., the screening and pathogen identification were conducted according to Office International des Epizooties (OIE) standardized protocols [20]. The presence of Chlamydophila spp. was investigated by real-time Polymerase Chain Reaction (PCR) directly in biological samples [21] and typing by the PCR-restriction fragment length polymorphism (RFLP) assay, targeting the 16S ribosomal gene [22]. For Mycoplasma spp., the PCR method described by van Kuppeveld et al. [23] was used. Virological analyses for detecting the more common pig pathogens were conducted using a panel of PCR methods including porcine reproductive and respiratory syndrome virus (PRRSV) (AgPath-ID™ NA and EU PRRSV Multiplex© Applied Biosystems), porcine circovirus type 2 (PCV-2) [24], porcine parvovirus (PPV) [25], and porcine circovirus type 3 (PCV-3) [26]. The presence of pestiviruses was determined by using a pan-pestivirus real-time RT-PCR [27], and, considering its limited capacity in detecting APPV, by a APPV-specific real-time RT-PCR [19]. In addition, all the tested samples were inoculated on cell cultures (primary embryonic swine kidney cells, swine alveolar macrophages, and monkey kidney cell line MARC-145), which allow the isolation of a broad range of swine viruses. The inoculated cell monolayers were observed daily for 5–7 d for the appearance of a cytopathic effect and then sub-cultured to the second passage, at which time they were independently tested using an “in-house” sandwich enzyme-linked immunosorbent assay (ELISAs) for the presence of pestivirus [28] and PRRSV antigens [29]. Only those samples that were positive for APPV at the initial screening test using the APPV-specific real-time RT-PCR were further subcultured to improve the chance to isolate the APPV until the fifth passage, at which time, even in the absence of a cytopathic effect, they were assessed again with the APPV-specific real-time RT-PCR.

### 2.2. Wild Boars

In total, 430 blood samples of wild boars, killed during the 2017–2018 hunting season, were collected in the framework of the Lombardy and Emilia Romagna wildlife monitoring plans for classical swine fever (CSF) and Aujeszky disease and transferred to IZSLER for examination. Serum samples were tested for antibodies against swine vesicular disease virus [30,31], encephalomyocarditis virus [32], glycoprotein E of Aujeszky’s disease virus [33], pestivirus (A–D) [34], swine influenza virus type A, subtypes H1N1, H1N2, and H3N2 [35,36], and finally for Brucella spp. (Svanovir Brucella–Ab C-ELISA©). Serological analyses were conducted with the methods currently in use at the IZSLER.

### 2.3. Identification and Genomic Characterization of Atypical Porcine Pestivirus (APPV)

For the investigation of the APPV genome, all samples, both the homogenates from fetal organs and the wild boar sera, were screened using the NS5B gene-specific real-time RT-PCR method [19]. Samples that tested positive were characterized by Sanger sequencing using RT-PCR that amplified a fragment of the NS3 region. The nucleotide sequences were aligned using the ClustalW method and compared with sequences present in GenBank [37] using MEGA6 software [38]. The maximum likelihood phylogenetic tree was constructed using IQ-tree software [39] by applying the TIM2+F+G4 model identified using ModelFinder selection [40].

## 3. Results

### 3.1. Pigs

**Figure 1:**
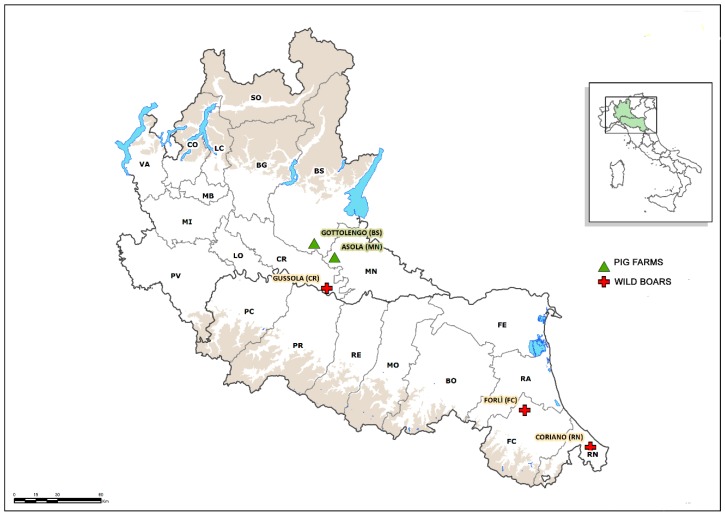
Geographical distribution of the pig farms and wild boar hunting sites where atypical porcine pestivirus (APPV) was identified.

The examination of the NS5B gene by real-time RT-PCR in the homogenized samples of pig fetuses identified two (0.6%) positive samples in two distinct farrow-to-finish farms, one from the province of Brescia in a pool containing the organs of three fetuses and the other from the province of Mantua in a pool containing the organs of two fetuses (Figure 1).

**Figure 2:**
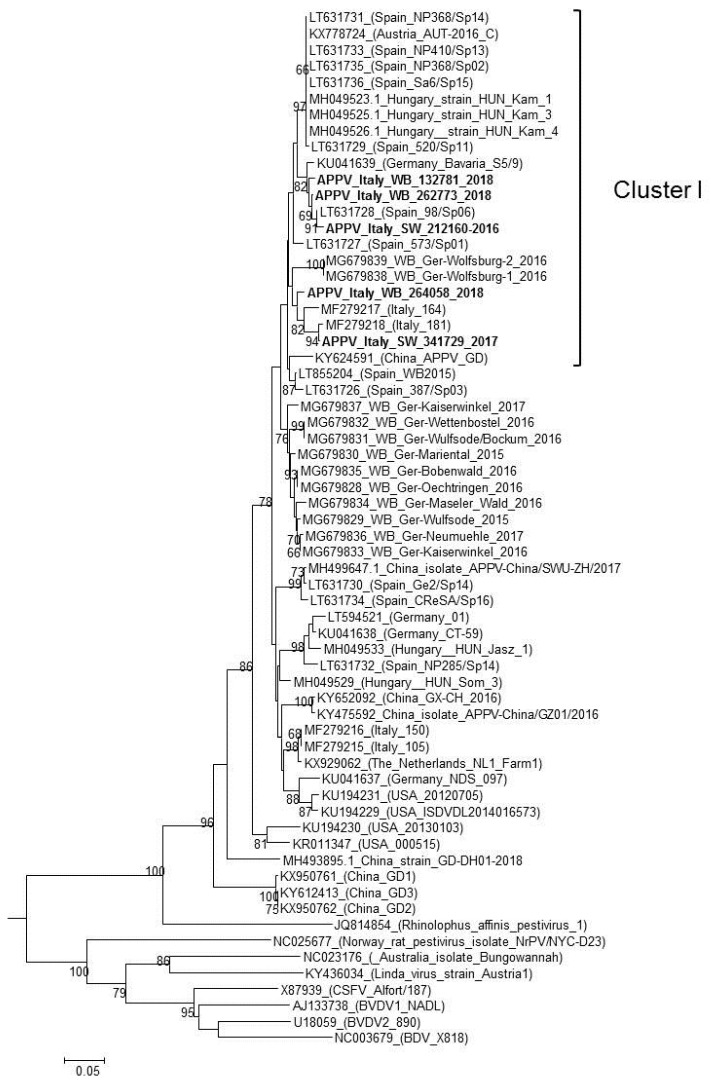
Phylogenetic tree based on a 645-nt fragment of the nonstructural protein 3-encoding region of the atypical porcine pestivirus (APPV) genome present in GenBank. A phylogenetic analysis using the maximum likelihood method including 1.000 bootstrap iterations was performed. Only bootstrap values ≥60 are indicated. Sequences in bold were generated in this study.

At necropsy, none of these fetuses showed internal macroscopic lesions, and bacteriological investigations consistently produced negative results. Moreover, the molecular examinations for PRRSV, PCV-2, PPV, and pan-pestivirus were all negative, and only the real-time PCR for PCV-3 produced a positive result from a pool of fetuses that originated from the province of Brescia. The two sequences obtained, APPV_Italy_SW_BS341729_2017 and APPV_Italy_SW_MN212160_2016, had a nucleotide similarity of 92.5% between them and clustered with APPVs previously identified in Europe. In particular, the phylogenetic tree constructed using the NS3 region (Figure 2) revealed that the two identified strains belong to distinct groups: (1) the APPV_Italy_SW_MN212160_2016 strain is related to both the 98/Sp06 strain identified in Spain in 2006 [16] (99.2% identity) and the German strain Bavaria S5/9 identified in 2015 [11] (96.8% identity); and (2) APPV_Italy_SW_BS341729_2017 forms a separate and closely related clade with two sequences identified in 2015 in pigs from Italy, Italy-164 and Italy-181 [15], with a nucleotide similarity of 95.5% and 99.2%, respectively.

Despite the positive results of the real-time RT-PCR test, virological examinations of the organs of swine fetuses by inoculating cell cultures up to the fifth passage produced consistent negative results. Consequently, we did not succeed in isolating any APPV strains.

### 3.2. Wild Boars

All of the wild boar samples examined in this study including those that tested APPV-positive, originated from hunting activity, and thus, they were considered healthy based on their behavior before shooting and the absence of lesions on carcasses examined at slaughter. Out of the 430 blood samples examined, three (0.69%), originating from the Cremona Province in Lombardy (APPV_Italy_WB_CR264058_2018) and from two neighboring provinces, Rimini and Forlì in Emilia Romagna (APPV_Italy_WB_RN262773_2018 and APPV_Italy_WB_FC132781_2018, respectively) (Figure 1), screened positive for APPV. In agreement with the genomic characterization of the strains identified in pigs, the sequences of those identified in wild boars could be divided into two distinct clusters. The first included the two strains detected in Emilia Romagna (APPV_Italy_WB _RN262773_2018 and APPV_Italy_WB _FC132781_2018) and the sequence identified in the pig farm from Mantua Province in Lombardy (APPV_Italy_SW_MN212160_2016), which had identities of 95.6% and 96%, respectively; plus the previously characterized strains BavariaS5/9 and Spain 98/Sp06, with which the WB strains had a higher nucleotide identity of 98–99.2%.

The second cluster included the APPV_Italy_WB_CR264058_2018, the APPV Italy_SW_ BS341729_2017 (96.4% identity), the Italian pig strains, Italy-164 and Italy-181, which had nucleotide of 96.4% and 96%, respectively, and the strains identified in 2016 in Lower Saxony, Germany (94.3% identity).

For the collateral serological examinations, all APPV positive samples were negative for all serological tests employed except for: (a) one serum (APPV_Italy_WB_CR264058_2018) that tested positive for anti-Brucella spp. antibodies and (b) one (APPV_Italy_WB_FC132781_2018) that tested positive for glycoprotein E antibodies to the Aujeszky’s disease virus.

The nucleotide sequences of the five APPV isolates were deposited in GenBank (NCBI) with the accession numbers MN736974–MN736978.

## 4. Discussion

The examination of 360 swine fetuses and 430 hunted wild boar blood samples identified APPVs in the area, two strains from the former and three strains from the latter. Based on the phylogenetic analysis performed, all five of these strains clustered with APPV strains previously identified in Spain and Germany. Although all of the sequences obtained can be grouped into the putative Cluster I described by Muñoz-González et al. [16], which should include viruses that have a common origin, the strains identified here could be further divided in two distinct sub-clusters.

Based on the absence of lesions on both fetuses and wild boars, we were keen to exclude a definite pathogenic role for APPV in these specific cases. However, it remains to be clarified whether the PCV-3 plus APPV (APPV_Italy_SW_BS341729_2017) co-infection, which was detected in the pool of fetuses originating from Brescia Province, may have had a synergistic effect in infected piglets, resulting in a clinical form characterized by abortion and natimortality. The very low prevalence of APPV found in the examined pig samples (0.6%) is largely different from the sole previous study on APPV in Italy [15], which reported a higher prevalence of viral detection (17.5%). This could be likely due to the sample types and selection, since we included in the survey only aborted pig fetuses with the aim of trying to establish if any correlation existed between the clinical case and the detection of APPV. A more specific monitoring program focused on the two APPV positive farms, and in general, to have systematically reported and analyzed cases of CT are anticipated in order to better define the prevalence of APPV in pig farms in Italy and clarify its effects.

Wild boars are susceptible to APPV infection, but their role in the epidemiology of the virus remains unknown. The low prevalence of APPV in the examined wild boar population is well correlated with the numbers reported by Colom-Cadena et al. [18] in Spain, but is in contrast to the high prevalence found in wild boars from northern Germany [17], where APPV seems to be endemic among wild boars in many areas. Considering the small number of strains isolated from the large geographical territory represented by the two regions (Lombardy and Emilia Romagna) and the lack of any particular geographic distribution of the clustered strains, it is impossible to draw epidemiological interpretations on APPV diffusion or on transmission between domestic pigs and wild boars.

## 5. Conclusions

APPV appears to be well established in the domestic swine populations of different countries in Europe, America, and Asia. Indeed, although identifying APPV has only become possible during the last few years due to the progressive refinement of diagnostic techniques, retrospective studies have indicated its circulation for many years. Based on the available genomic characterization data in the literature and the prevalence in domestic pigs, APPV exhibits a high genetic diversity among viral strains detected in different countries and tends to form independent clusters according to geographic locations. This study confirms the presence and distribution of APPV in populations of domestic and wild pigs present in the Lombardy and Emilia Romagna regions of Italy, which are under the jurisdiction of IZSLER. The high homology levels with strains identified in Germany and Spain reinforce the hypothesis that Italian strains have a European origin, and they confirm the likely determining role in the spread of infection being the commercial trade in pigs among different countries. The detection of new pestiviruses indicates the need to monitor for their presence and distribution using a systematic surveillance and diagnostic approach. In fact, the accurate and constant characterization of circulating strains is necessary to update the serological and virological tests, which in turn may be used to collect more detailed epidemiological information regarding APPV such as routes of entry and dissemination, and genetic evolution.
